# Food-Web Structure in Relation to Environmental Gradients and Predator-Prey Ratios in Tank-Bromeliad Ecosystems

**DOI:** 10.1371/journal.pone.0071735

**Published:** 2013-08-14

**Authors:** Olivier Dézerald, Céline Leroy, Bruno Corbara, Jean-François Carrias, Laurent Pélozuelo, Alain Dejean, Régis Céréghino

**Affiliations:** 1 EcoFoG, Ecologie des Forêts de Guyane, CNRS UMR 8172, Kourou, France; 2 AMAP, botAnique et bioinforMatique de l’Architecture des Plantes, IRD UMR 123, Montpellier, France; 3 LMGE, Laboratoire Microorganismes: Génome et Environnement, Université Blaise Pascal, CNRS UMR 6023, Aubière, France; 4 EcoLab, Laboratoire Ecologie Fonctionnelle et Environnement, Université Paul Sabatier, Toulouse, France; 5 EcoLab, Laboratoire Ecologie Fonctionnelle et Environnement, CNRS UMR 5245, Toulouse, France; University of Arizona, United States of America

## Abstract

Little is known of how linkage patterns between species change along environmental gradients. The small, spatially discrete food webs inhabiting tank-bromeliads provide an excellent opportunity to analyse patterns of community diversity and food-web topology (connectance, linkage density, nestedness) in relation to key environmental variables (habitat size, detrital resource, incident radiation) and predators:prey ratios. We sampled 365 bromeliads in a wide range of understorey environments in French Guiana and used gut contents of invertebrates to draw the corresponding 365 connectance webs. At the bromeliad scale, habitat size (water volume) determined the number of species that constitute food-web nodes, the proportion of predators, and food-web topology. The number of species as well as the proportion of predators within bromeliads declined from open to forested habitats, where the volume of water collected by bromeliads was generally lower because of rainfall interception by the canopy. A core group of microorganisms and generalist detritivores remained relatively constant across environments. This suggests that (i) a highly-connected core ensures food-web stability and key ecosystem functions across environments, and (ii) larger deviations in food-web structures can be expected following disturbance if detritivores share traits that determine responses to environmental changes. While linkage density and nestedness were lower in bromeliads in the forest than in open areas, experiments are needed to confirm a trend for lower food-web stability in the understorey of primary forests.

## Introduction

Biodiversity is not only the sum of coexisting species, it is also the diversity of interactions that connect these species. It is widely acknowledged that linkage patterns among species, rather than species richness alone, are a key component of the diversity-stability relationship [1]. For instance, for a fixed number of species, food-web persistence and robustness (two concepts linked to network stability) are believed to increase with connectance, the proportion of all possible interactions that are realized [2]. A rich body of literature has focused on the relationship between network structure and dynamics [3], and on how different types of interactions (either antagonistic or mutualistic) affect network properties [4]. Most studies however focused on network structure regardless of variation in the abiotic environment. Consequently, our understanding of the environmental determinants of network structure lags behind the increasingly vast knowledge of species richness- and interactions-mediated patterns [5], [6]. Identifying linkages between ecological networks and physical environments could be relevant to predict the extent to which entire communities will respond to increasing environmental disturbances (e.g., climate change, habitat fragmentation, overharvesting, introduction of invasive species, pollution).

On a local scale, both the spatial/temporal changes in habitat conditions and species traits regulate community structure [7], thereby affecting ecosystem functions. For instance, the nature and extent of the riparian vegetation determines food quality and availability in still [8] and running waters [9], thus influencing the functional feeding group composition of macroinvertebrate communities [10]. However, we know little about how linkage patterns among species (food-web topology) change against a background of environmental gradients over broad, regional scales. Most of what we know about environmental determinants of food-web structure comes from experiments in single locations [11], on a target fraction of the food web (e.g., vertebrates, macro- and micro-invertebrates excluding components such as microorganisms, bacteria, flagellates, rotifers and viruses; [12]).

It has proven challenging to study the effects of both abiotic and biotic factors on food webs. High species diversity and population densities in large ecosystems preclude the accurate characterization of trophic links (notably in continuous habitats), and because it is often difficult to manipulate entire ecosystems. To tackle these issues, we focussed on small, spatially discrete food webs that naturally span a broad range of environmental gradients. Bromeliaceae are flowering plants represented by 59 genera and some 3140 species native mainly to the Neotropics [13]. The interlocking leaves of tank-forming bromeliads form wells that collect water, leaf litter and other organic detritus. The detritus that enter the tank constitute the main source of nutrients for the aquatic food web [14]. The aquatic communities inhabiting tank-bromeliads provide excellent opportunities to study food-web structure because they contain several trophic levels (from bacteria to predatory macroinvertebrates; [15]) and can be exhaustively sampled [16].

The aim of this study was to determine whether spatial patterns in food-web structure can be predicted from a small set of environmental factors and/or the richness of predators relative to their prey. To address this question, we sampled 365 tank-bromeliads in a wide range of environments in French Guiana (plantations, pioneers growths, rock savannah, primary forest) and drew the 365 corresponding food webs using the gut contents of invertebrates as well as observations of predator-prey encounters. Previous studies on tank-bromeliads concluded that detrital resources at the base of the food web, understorey light environments (energy available for algal production), habitat size and predation play key roles in shaping aquatic community composition [17], [18]. Detritus constitute the main source of energy for aquatic bromeliad communities, however, the high algal biomass found in sun-exposed bromeliads [19] may provide a complementary non-detrital resource to the upper trophic levels. If (i) species richness and abundance increase with bromeliad (habitat) size [20]–[24], and (ii) bromeliads from open area benefit from both detrital and non-detrital resources and understorey bromeliad foodwebs are solely supported by detrital matter [15], then, for a given habitat size, the diversity of invertebrate functional feeding groups should increase from forest to open areas. One may expect shifts in food-web connectance, linkage density, and/or nestedness as species with particular traits are replaced or complemented by species with other traits when shifting from forest understorey to open areas. Conversely, if the resource that supports food webs does not differ from forest to open areas, then one should not observe significant shifts in food-web structure and functions. To test these hypotheses, we used Linear Mixed Effect modelling to analyse patterns of community diversity and food-web topology in relation to key environmental variables and predator:prey richness ratios.

## Materials and Methods

### Ethics Statement

This study was conducted according to relevant national and international guidelines. Sample collections necessary to scientific research were authorized by the French *Office National des Forêts* (ONF) provided that their impact upon the environment is considered negligible.

### Study Area, Bromeliads and Environmental Variables

The study was conducted in French Guiana, from March 2006 to October 2011. The climate is tropical moist with 3,000 - 3,400 mm of yearly precipitation mainly distributed over 280 days. There is a major reduction in rainfall between September and November and another shorter and more irregular dry period in March. The maximum monthly temperature averages 33.5°C (32.1–35.8°C), and the monthly minimum averages 20.3°C (19.7–21°C).

**Figure 1:**
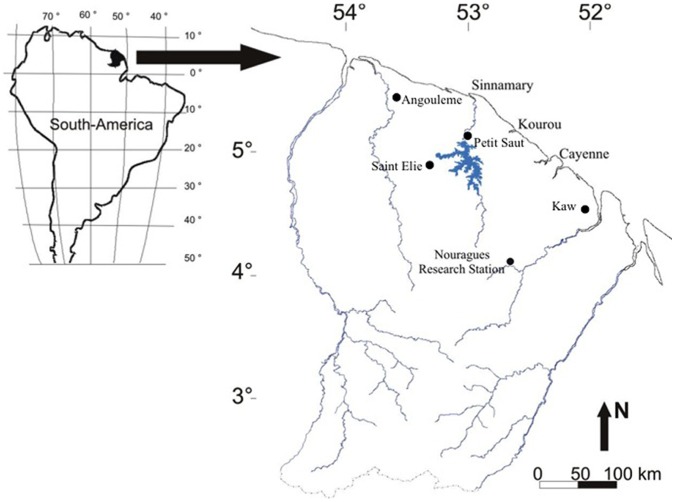
Distribution of the sampling locations in French Guiana (see Table 1).

We selected five sampling localities distributed across a south-east to north-west range (Fig. 1) and sampled 365 bromeliads (i.e. 365 food webs) in the understorey of primary and transitional forests, in pioneer growths, in a rock savannah, and in plantations. The main habitat characteristics of tank-bromeliad species found in five vegetation types at five localities (hereafter “vegetation types”, within localities) are summarized in Table 1. Further descriptions of the Nouragues (sampling period in April 2006), Petit-Saut (March 2009) and Kaw (Oct. 2008) localities and their bromeliads can be found in [15], [22], [25]. Saint-Elie (Oct. 2007) and Angoulème (April 2010) are *Citrus* plantations. Both epiphytic bromeliads and those that had taken root on the ground were included in the study.

**Table 1:** Main habitat characteristics (mean±standard error) of tank-bromeliad species found in five vegetation types.

Locality	Vegetation types	Species	N	IR	WV	FPOM	#Taxa
Nouragues	Rock savannah	CB	29	73.3±2.4	40.62±3.75	0.74±0.08	10.55±0.15
		AA	31	66.97±2.73	949.23±102.64	10.84±1.26	15.87±0.42
	Transitional forest	VP	30	25.12±0.29	73.2±9.11	1.7±0.28	10.3±0.35
		AB	26	25.69±0.47	137.85±21.1	1.08±0.25	11.23±0.32
	Primary forest	VS	26	18.75±0.4	48.54±5.03	4.29±0.42	10.35±0.25
		GL	19	15.9±0.6	17.46±2.53	1.04±0.16	9.21±0.29
Petit-Saut	Pioneer growth	AM	63	39.33±2.63	84.38±10.1	6.16±1.26	12.49±0.28
	Primary forest	VS	34	16.64±0.38	26.18±3.84	3.46±0.71	10.68±0.23
Kaw	Pioneer growth	AM	45	35.1±2.98	92.46±11.82	4.3±0.68	11.78±0.28
Angoulème	*Citrus* plantation	AM	35	33.03±2.95	31.01±4.54	2.32±0.31	11.8±0.34
Saint-Elie	*Citrus* plantation	AM	27	32.07±1.3	56.41±8.29	4.58±0.86	14.22±0.36

CB: *C. berteroniana*; AA: *A. aquilega*; VP: *V. pleiosticha*; AB: *A. bromeliifolia;* VS: *V. splendens*; GL: *G. lingulata*; AM: *A. mertensii.* N = number of plants sampled, IR = incident radiation (%), WV = water volume extracted (mL), FPOM = fine particulate organic matter (mL after decantation in test-tubes), #Taxa = number of taxa per food web.

In order to prevent seasonality effects (i.e., dry *vs* rainy season) on water volumes and species abundance, the sampling took place at the transition between rainy and dry seasons, where invertebrate abundances reach a peak. We also sampled tank-bromeliads that were full of water. Species occurrence was not an issue as bromeliad invertebrates have fast life cycles (<2 months) with overlapping cohorts. To characterize habitat size, we emptied the wells in each plant by sucking the water out (see invertebrate sampling) and recorded the corresponding volume of water (WV, mL). The amount of fine particulate organic matter (FPOM; 1000 to 0.45 µm in size) was expressed as preserved volume (mL) after decantation in graduated test-tubes [12]. Finally, percentages of total incident radiation (IR) above the bromeliads were calculated using hemispherical photographs and an image processing software (Gap Light Analyzer 2.0; [26]), as described in [23]. In this study, we consider that all bromeliads with an IR below 50% were located in partially shaded areas or forested environments while higher percentages defined sun-exposed areas.

### Aquatic Invertebrate Communities

For both ethical (extensive sampling could destroy local populations) and legal reasons (the Nouragues Research Station is located in a nature reserve), we decided to use a non-destructive sampling technique for all studied bromeliads. To sample the water retained in the tanks, we used 5-mL and 10-mL automatic micropipettes with the end trimmed to widen the orifice. We homogenized the water within leaf wells by sucking in and out with a pipet, before sucking out the content. Although less efficient than plant dissection, we and other researchers have already successfully used this technique [24], [27]. It was consistently used for all of the samples and most of the water (>95%) was collected. Both early and late instars of prey and predator invertebrates were captured, so we were confident that our technique was efficiently implemented. The samples were preserved in the field in 4% (final concentration) formalin. Invertebrates were sorted in the laboratory and preserved in 70% ethanol. They were identified to genus, species or morphospecies and enumerated (species lists in [21], [24]). Species abundance data (individuals per plant) were used to calculate evenness (Simpson index) and entropy (Shannon-Weaver index) for each invertebrate community. The Simpson index was calculated as *D = *Σ p_i_^2^. The Shannon-Weaver index (hereafter Shannon index) was defined as *H = *−Σ p_i_ log(b) p_i_, where p_i_ is the proportional abundance of species *i* and *b* is the base of the logarithm (natural logarithm in this case). Species richness and abundance being components of these two indices, they were not considered alone in subsequent modeling. Macroinvertebrate taxa were partitioned into predators (carnivorous species which attack and consume living macroinvertebrates) and prey (species which sift fine particulates and microorganisms from the water column and/or gather FPOM and associated microorganisms from the accumulated debris), and these categories were used to calculate Predator:Prey Richness ratios (number of predatory taxa/number of prey taxa, hereafter PPR).

### Food Web Characterization

The diet of the various invertebrate species that make up food webs was determined by dissecting the entire guts. Twenty to 50 individual guts from each taxon were dissected across both localities and vegetation types in order to encompass the variability in diets of omnivores and predators. The gut contents of predators were placed into a drop of water on a glass slide, spread out, and analysed using a binocular microscope (Leica® MZ 12.5) and an Optiphot-2 Nikon® microscope whenever necessary. Most of the prey items could be identified from the guts of predators by comparing the chitinous parts (head capsules or legs, setae…) with specimens of bromeliad invertebrates archived in our collection (Univ. of Toulouse III). Only the gut contents of piercers (Heteroptera Veliidae, Diptera Tabanidae) could not be identified visually; in this case, we relied on observations of arranged encounters in test tubes.

The microorganisms found in the guts of detritivores were identified to a coarse taxonomic level using an Optiphot-2 Nikon microscope at x600 magnification. Determinations were based on earlier descriptions ([28], [29]), and on recent microbiological samples (Carrias J-F, unpublished data). The presence of particulate organic matter was also recorded in the gut contents, and we used an ocular micrometer to distinguish fine particulate organic matter (FPOM, 1000 to 0.45 µm in size) from coarse particulate organic matter (CPOM, >1000 µm). Gut contents and other observations were used to build 365 interaction matrices (one per bromeliad) that described 365 “connectance webs” *sensu* [30] (see Fig. 1).

Three topological descriptors were used to describe food webs: connectance, linkage density, and nestedness. In the literature, two measures of the connectance are widely used: connectance and normalized connectance. Both refer to the proportion of all possible interactions that are realized, but normalized connectance is rather used for food webs comprising less than 20 species [1]. However, in this study, the two measures were strongly correlated (Pearson’s correlation coefficient = 0.966, *p*<0.0001) and we therefore used the connectance as defined by [31]:
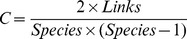



Linkage density was calculated as the ratio between the number of links and the number of species. Levels of nestedness were expressed as matrix temperatures, with values ranging from 0 (perfectly nested) to 100 (see [32]) were calculated from the 365 interaction matrices (binary data) using the BINMATNEST3 program [33]. Departure from random was tested against a number of 1000 random matrices using the most conservative null model in BINMATNEST3 (namely “model 3″). A matrix temperature is affected by both its structure (the fill) and its size (the number of rows and columns). The extent to which the observed temperature departs from random depends on how random matrices are built. Unlike the null models 1 and 2, the model 3 is a packing algorithm that generates random matrices that are less influenced by the structure of the input matrix, thus allowing for comparisons with other studies [33]. More specifically, the null model 3 generates random matrices where each cell has a probability of containing a “1″ equal to the averaged probability of occupancy of rows and columns of the input matrix. Therefore, the fill of each cell depends on both the fraction of “1s” in rows and columns in which the cell is included.

### Data Analysis

To analyze the relationships between food-web descriptors, environmental variables and PPRs, we used linear mixed effect modeling. All variables were log-transformed to fit a normal distribution. Because a given vegetation type was found at only one locality, we qualified this structure as the “vegetation type nested within locality”. In contrast, a given bromeliad species could be sampled at many localities or many vegetation types, therefore the variable “bromeliad species” was not nested within the former or the latter. Since “locality”, “vegetation type”, “vegetation type nested within locality” or “bromeliad species” could be included as random factors, we performed a model selection based on the more conservative Bayesian Information Criterion (BIC) of the full models (models considering all environmental variables) for each dependent variable. Then, for a selected random factor (the remaining random effects being dropped from the model), the relationship between dependent and independent variables were explored using a stepwise backwards removal procedure and only the final models containing significant variables were presented. Departures from homoscedasticity and the normality of the residual errors were evaluated graphically for each final model. All statistical analyses were evaluated under a 95% confidence level and were conducted using R software V. 2.14.1 [34] and the associated packages for Linear Mixed Effect Modeling (nlme, lmer4).

## Results

### Abiotic Environments and Food-web Composition

There were large differences in WV and FPOM among bromeliad species, even at a given vegetation type (Table 1). The two bromeliads growing in rock savannah (*A. aquilega* and *C. berteroniana*) showed the highest and lowest mean values for FPOM (10.84 mL *vs*. 0.74 mL, respectively), and the highest and one of the lower mean values for WV (949.23 mL *vs*. 40.62 mL, respectively) compared to the others tank-bromeliad species. Thus, even though the understorey of primary forests consistently received lower incident radiation (i.e. *V. splendens* and *G. lingulata*) and higher litter inputs than sun-exposed areas, the amount of FPOM inside the tank of bromeliads from primary forests was not necessarily higher than those from rock savannah. However, bromeliads from primary forests had higher FPOM:WV ratios (0.05–0.09 mL FPOM/mL WV) than bromeliads from open areas (0.01–0.02 mL FPOM/mL WV).

**Figure 2:**
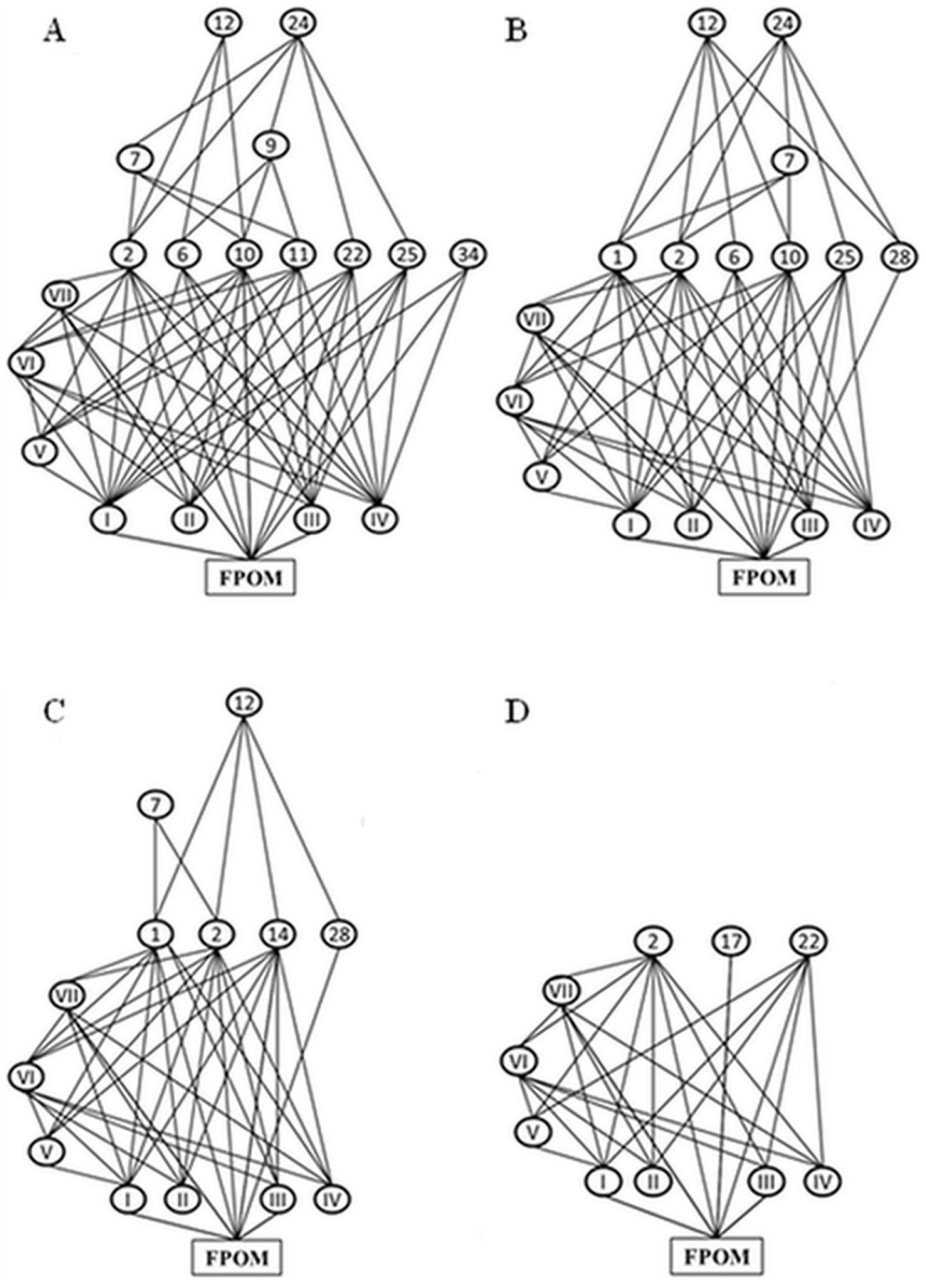
Examples of connectance webs under two contrasted environmental conditions: open areas (A, B) and forest areas (C, D). The upper trophic level (predators) is at the top of the graphs, and the lower (algae, detritus) at the bottom. Numbers and abbreviations are for: *Wyeomyia* spp. (1; filter-feeder); *Culex* spp. (2; filter-feeder); *Forcypomiinae* sp2 (6; filter-feeder); *Bezzia* sp. (7; predator); Tanypodinae (9; predator); Chironominae (10; detritivore); Tanytarsinii (11; detritivore); *Corethrella* sp. (12; predator); *Telmatoscopus* sp1 (14; detritivore); Limoniinae (17; detritivore-shredder); *Cyphon* sp. (22; shredder); Coenagrionidae (24; predator); *Aulophorus superterrenus* (25; detritivore); Hydracarina (28; detritivore); *Elpidium* sp. (34; detritivore-scraper); Bacteria (I); Cyanobacteria (II); Fungi (III); Algae (IV); Flagellate (V); Ciliate (VI); Rotifera (VII); Fine Particulate Organic Matter (FPOM).

Food webs comprised 8 to 20 taxa (median = 11 taxa), including macroinvertebrates, rotifers, and miscellaneous microorganisms (bacteria, cyanobacteria, fungi, algae, heterotrophic flagellates, and ciliates) identified in the gut contents. The predators belonged to the Diptera Culicidae (*Toxorhynchites purpureus*), Corethrellidae (*Corethrella* sp.) and Tabanidae, and to the Odonata (one unidentified Coenagrionidae species). Detritivores mostly consisted in Diptera Culicidae (*Culex* spp. and *Wyeomyia* spp.), Limoniinae, Chironomidae (*Tanytarsus* sp.) and Psychodidae (*Telmatoscopus* spp.). The list of taxa and the corresponding functional groups is provided in Fig. 2.

### Model Selection and Influence of Abiotic and Biotic variables on Food Webs

All random factors were selected in at least one model (Table 2), suggesting that different spatial scales could provide complementary explanations to the observed pattern. More specifically, “vegetation type” and “bromeliad species” were included as random factors in the Simpson index (BIC = −314.16) and connectance models (BIC = −1108.648), respectively. “Locality” was selected in the Shannon index, linkage density and nestedness models (BIC = 21.83, BIC = −586.99, and BIC = −546.25, respectively). The subsequent stepwise backward removal procedures for each food-web descriptor were thus performed using their selected random factors. The computation of likelihood ratios demonstrated that we could not discriminate the model with “locality” from the one with “vegetation type” as random factor for the Shannon index model (L = 0.034, *p* = 0.92). Therefore, the variable selection for the Shannon index model was also performed with “vegetation type” as random factor. In general, the inclusion of random factors with higher BIC values (e.g., “vegetation type”, “vegetation type nested within locality” or “bromeliad species”) only slightly affected the outcomes of the models in terms of significant and non-significant interactions among variables. More specifically, interactions that were barely significant with the selected random factor became non-significant when other random factors were included.

**Table 2:** Bayesian Information Criterion (BIC) of the full models with four different random effects for each food-web descriptor.

		Random effects			
Models	Vegetation type	Locality	Locality/Vegetation type	Bromeliad species	
Shannon-Weaver’s index	21.87	**21.83**	25.79	31.69	
Simpson’s index	−**314.16**	−310.39	−308.73	−310.19	
Connectance	−1102.57	−1090.4	−1097.89	−**1108.65**	
Linkage density	−584.88	−**586.99**	−582.41	−564.51	
Nestedness	−544.09	−**546.25**	−541.21	−527.14	

The random effect ‘Locality/Vegetation type’ means that the vegetation types are nested within the localities. Bold characters highlight the lowest BICs.

The Shannon and Simpson indices were both positively correlated with WV (*p*<0.0001) and with PPR (*p = *0.0009 and *p* = 0.0027, respectively; Table 3), showing that invertebrate community diversity increased with habitat size and the relative number of predators. Moreover, these two models showed a negative interaction between FPOM and PPR (*p* = 0.036 and *p* = 0.047, respectively). However, when “vegetation type” was included as random factor in the Shannon index model, both water volume and PPR remained significant but the FPOM:PPR interaction was not significant (*p* = 0.081). Last, the variable “IR” was not significantly correlated with the Shannon index, whatever the random factor included (*p* = 0.6–0.75). Finally, the two negative interactions IR:WV and PPR: FPOM become significant (*p* = 0.046 and *p* = 0.014, respectively) for the Simpson index when “Locality” was included as a random factor.

**Table 3:** Models evaluating the patterns of community diversity (Shannon’s entropy and Simpson’s evenness) and food-web structure (connectance, linkage density, nestedness) in relation to environmental variables and their interactions.

Fixed Effects	Estimate±SE	t-value	df	*p*	RandomEffects
**Shannon**					Locality
Intercept	0.296±0.068	4.305	356	<0.0001	
Slope					
WV	0.091±0.011	7.72	356	<0.0001	
FPOM	0.044±0.025	1.773	356	0.077	
PPR	0.268±0.08	3.357	356	0.0009	
FPOM:PPR	−0.126±0.059	−2.103	356	0.036	
**Simpson**					Vegetationtype
Intercept	0.226±0.036	6.157	352	<0.0001	
Slope					
WV	0.04±0.008	4.889	352	<0.0001	
FPOM	0.02±0.016	1.226	352	0.22	
PPR	0.15±0.049	3.025	352	0.0027	
FPOM:PPR	−0.074±0.037	−1.992	352	0.047	
**Connectance**					Bromeliadspecies
Intercept	0.60±0.017	35.018	355	<0.0001	
Slope					
WV	−0.007±0.002	−2.671	355	0.0079	
FPOM	−0.007±0.003	−2.07	355	0.039	
PPR	−0.14±0.009	−15.039	355	<0.0001	
**Linkage** **Density**					Locality
Intercept	1.136±0.102	11.117	354	<0.0001	
Slope					
IR	0.079±0.027	2.85	354	0.0046	
WV	0.141±0.029	4.77	354	<0.0001	
FPOM	−0.087±0.047	−1.854	354	0.064	
PPR	−0.186±0.019	−9.365	354	<0.0001	
IR:WV	−0.028±0.008	−3.4	354	0.008	
IR:FPOM	0.028±0.013	2.124	354	0.034	
**Nestedness**					Locality
Intercept	3.302±0.107	30.664	355	<0.0001	
Slope					
IR	0.087±0.029	3.014	355	0.0028	
WV	0.106±0.025	4.126	355	<0.0001	
FPOM	0.02±0.007	2.619	355	0.0092	
PPR	−0.237±0.021	−11.204		<0.0001	
IR:WV	−0.019±0.008	−2.708	355	0.0071	

IR = %incident radiation, WV = water volume (mL), FPOM = fine particulate organic matter (mL), PPR = Predator:Prey Ratio (see text). Only variables and interactions with *p*<0.05 are interpreted as statistically significant and presented in the table.

Connectance showed a negative and significant correlation with WV and PPR (*p* = 0.0079 and *p*<0.0001, respectively), and there was a trend for decreasing connectance with increasing FPOM amount (*p* = 0.039; Table3). Incident radiation was not significantly correlated with connectance (*p* = 0.24). When “Locality” was included, the two negative interactions IR:WV and WV:FPOM turned to be significant (*p* = 0.0002 and *p* = 0.009, respectively) in the connectance model. Both the linkage density and nestedness models showed a significant negative interaction between WV and IR (*p = *0.008 and *p* = 0.0071, respectively). Linkage density and nestedness were positively correlated with WV and IR, and negatively correlated with PPR (Table 3). Linkage density was positively correlated with the IR:FPOM interaction (*p = *0.034), but did not correlate with FPOM alone (*p* = 0.064). Finally, levels of nestedness (*N*) ranged from 21.54 to 49.79 (median *N = *39.98). Overall, 44.4% of our interaction matrices were significantly different from the null matrices obtained at random (*p = *0.0001 to 0.99, median *p = *0.08). Nevertheless, this percentage increased with the number of taxa; for instance, 66.1% and 88.8% of all of the interaction matrices comprising ≥13 and ≥17 taxa, respectively, were significantly different from random.

## Discussion

Based on an unprecedented number of replicates under natural conditions, our models have proven informative in assessing whether environmental factors and biological interactions explain some patterns of food-web structure. Overall, variations in food-web structure across environments and spatial scales were mostly due to interactions between habitat size and the distribution of predators (Kendall’s test, positive correlation between WV and PPR, ρ = 0.179, *p*<0.0001). There was however a trend for more feeding links in open areas compared to forest understorey. Finally, a core of generalists remained relatively constant across large regional scales, so that patterns of food-web connectance, linkage density and nestedness were mostly related to the addition or loss of predators.

Water volume was one of the most significant variables in all models, suggesting that habitat size was the primary factor controlling species composition and abundance patterns and, subsequently, food-web structure. Larger habitats are more easily colonized by immigrants, resulting in positive species-area relationships [35]. An increase in habitat area also fosters functional diversity [36], while allowing for a better partitioning of food resources by coexisting species [37]. Hence, at any given locality, both the number of taxa and individuals per plant increased with habitat size (there was a positive correlation between WV and PPR, Kendall’s test = 0.179, p<0.0001), which is consistent with previous findings on bromeliad ecosystems [21] and small wetlands in general [38]. However, there was a trend for bromeliad with higher incident radiation to accumulate more water (positive WV to IR relationship, r = 0.32, *p*<0.05, log-transformed data) certainly because in open areas there are fewer overhanging trees to keep most of the rain from reaching the bromeliads. Indeed, based on measures made in the understorey of the primary forest in Petit-Saut, we estimated that 30–38% of the rain is intercepted by the canopy. Hence, for a given bromeliad species and size, the containers hold more water at sun-exposed areas. Nevertheless, the complex interaction between habitat size and incident radiation (significant interaction between IR and WV in the linkage density and nestedness models, but not in the connectance model) precludes a precise distinction of their relative impacts on food webs. We cautiously suggest an indirect effect where IR mediates food-web features through bromeliads’ carrying capacity for aquatic invertebrates.

In aquatic ecosystems, detrital inputs form a strong trophic link between plant production, decomposer microorganisms, and larger metazoans [39]. In tank-bromeliads, detritus constitutes the main source of nutrients for the aquatic food web [14]. Debris-chewing invertebrates process incoming litter. Small particles, including faeces, are then washed into the plant pools where the FPOM is further processed in the gut of invertebrate collectors and filterers. Dead organisms, litter, and faecal particles, which collect in the leaf bases, are utilized by bacteria and other microorganisms. The nature and extent of the vegetation that surrounds these systems was therefore expected to have a strong influence on food-web structure through food quality and availability. On one hand, algae were found to account for more than 30% of the total microbial diversity and biomass in sun-exposed bromeliads [17], [19], which usually receive lower amounts of leaf litter [15]. We suggest that such a complementary “green” food web could contribute to reducing functional redundancy in FPOM-poor systems. Overall, there was a positive correlation between WV and FPOM (Kendall’s test = 0.349, p<0.0001). However, higher FPOM concentrations (i.e. the amount of FPOM in relation to WV) decreased the amount of open water inside of the tank, which had a negative effect on aquatic organism diversity. High FPOM concentrations may have the effect of clogging habitats in small freshwater ecosystems and, to a certain extent, space availability [21]. Since FPOM was not consistently significant in our models, we assume that resource availability is not a limiting factor in bromeliad systems. However, the influence of resource quality (detritus *vs*. algae) on individual food apportionment should deserve more attention in future studies.

The PPR was a highly significant variable in all models. Connectance, linkage density and nestedness (matrix temperature) significantly decreased with increasing proportions of predators. A decrease in connectance and linkage density can be due to a gain of specialists and/or a loss of generalists [1]. In our study, predators were generalist species in that they fed on numerous prey species and multiple trophic levels, but they could be considered as “node specialists” since they interacted mainly with their prey while prey established links with both basal species (algae, rotifers, ciliates…) and predators. The extent to which predators are specialized on specific prey plays a great role in generating patterns of community diversity from site-to-site to large regional scales [40], [41]. We found that the Shannon and Simpson indices (entropy and evenness, respectively) were positively correlated with the PPR at relatively small scales (from individual bromeliads to local scales), but overall, bromeliad food webs comprising less than 13 species were primarily composed of microorganisms and primary consumers, and were characterized by high levels of diffuse interactions where all species were more or less closely linked to each other. Indeed, a core of highly-connected species remained relatively constant at lower trophic levels (see also [42]), both in terms of species identity and ecological function, between individual bromeliads and from open to closed areas. For instance, the dominant generalist detritivores belonging to the Culicidae (*Wyeomyia* sp., *Culex* sp., and *Anopheles* sp.), Chironomidae and Oligochaeta were found in 310, 215 and 133 plants out of 365 respectively. The main top-predators, namely Corethrellidae, Ceratopogonidae and *Toxorhynchites* sp., occurred in 122, 105 and 83 plants respectively. It is likely that this highly-connected core ensures food-web stability and key ecosystem functions (e.g., decomposition, nutrient cycling) across environments. If detritivores share traits that determine responses to environmental changes in any given area (e.g., resistance to dessication, dispersal ability), then larger deviations in food-web structures can be expected following a major disturbance. Beyond 13 species, community diversity and food-web nestedness increased as a result of the addition of specialist predators and generalist detritivores. Our observations suggest that predators could enter the food webs at an average water volume of 49.9 mL, which corresponded to 1 predator species for 3 prey species on average. Regardless of the locality, larger bromeliad hosted a higher proportion of predators. In addition to their intrinsic value for biological diversity, predators (i.e., *Toxorhynchites* sp., *Corethrella* sp. and Coenagrionidae) thus played a key role in generating food-web patterns. This assumption is supported by the fact that, whilst predators are less frequent than detritivores, the predator:prey ratio was a significant variable in all three food-web topology models (connectance, linkage density and nestedness). We suggest that the addition of species at higher trophic levels contributes to departure from random interactions through the partition of prey resources between coexisting predators. Our results are also in line with studies which demonstrated that specialized species tend to be dependent on a core of densely connected generalist species, and that natural communities display non-random interaction patterns [32], [43]–[45]. Therefore, although we relied on unweighted trophic links to analyse spatial patterns in food-webs, this study provides strong empirical support on how food-web structure change along environmental and predator richness gradients. We conclude that at the bromeliad scale, habitat size determines the number of species that constitute food-web nodes and the proportion of predators (positive relationship), and subsequently, food-web topology. Hence, the number of species as well as the proportion of predators within assemblages globally decline from open to closed (forest) habitats, where the volume of water collected by bromeliads is generally lower because of rainfall interception by the canopy. The extent to which stability is related to linkage patterns (notably nestedness) in networks such as food webs remains however unclear and obviously deserves further research [46]. While linkage density and nestedness were lower in forested areas, experimental research is now needed to confirm a trend for lower food-web stability in the understorey of primary forests.
